# Retirement and Household Expenditure in Turbulent Times

**DOI:** 10.1007/s10834-022-09884-7

**Published:** 2023-01-12

**Authors:** Ioannis Laliotis, Mujaheed Shaikh, Charitini Stavropoulou, Dimitrios Kourouklis

**Affiliations:** 1 University of Peloponnese, Tripoli, Greece; 2 Department of Economics, City University of London, London, UK; 3 Global Labor Organization (GLO), Essen, Germany; 4 Hertie School of Governance, Berlin, Germany; 5 City University of London, London, UK; 6 The Office for Health Economics, London, UK

**Keywords:** Retirement, Household expenditure, Crisis, Greece, J26, D14, C26

## Abstract

We examine how expenditure changes at retirement during an institutionally and economically uncertain period when a series of pension reforms and cuts were implemented. Overall, we fail to confirm that consumption declines at retirement using data from Greece (2008–2018). Any estimated declines come from turbulent years when major pension cuts were applied. Expenditure drops at retirement were due to pension income shocks, especially for those who were particularly dependent on pension income. Further checks support the presence of an income shock mechanism for retirees who are relatively more treated during the crisis sub-period. Given an aging population and the ongoing global turbulence, our results offer valuable insights.

## Introduction

Many studies have provided evidence of declines of expenditure at retirement. Yet, those studies have analysed the relationship during periods of financial stability. There is hardly any empirical evidence on how household expenditure behaves during periods of financial crisis, although it has been widely acknowledged that such periods exert considerably adverse effects on pensions, as income sources, as well as the way they are spent (Grafova et al., 2020; Impavido & Tower, 2009). In this paper, we examined how expenditure behaves at retirement during turbulent times using household-level data, and examined the role of pension cuts during a severe financial crisis in explaining the drop in expenditures at retirement. We focused on Greece, the European country that was hit the hardest following the 2008 global financial crisis. Therefore, our results can provide valuable insights for policy making during uncertain periods. Moreover, as Greece has one of the highest shares of populations aged over 65 among the OECD countries (OECD, 2020), our study is also relevant to policies focusing on this growing part of the population.

In general, expenditure decreases at retirement are not consistent with predictions of the standard life-cycle model, in which households smooth out their marginal utility of consumption in expectation. While many studies provide evidence of such declines, labelled as the “retirement-consumption puzzle” (e.g., Banks et al., 1998; Bernheim et al., 2001; Dong & Yang, 2017), several others argue to the contrary and try to attribute them to factors that fall well within the scope of the life-cycle model (Aguiar & Hurst, 2005, 2013; Hurd & Rohwedder, 2003). For example, declines in spending at retirement due to a reduction in lifetime resources from unexpected events such as health shocks or involuntary transitions to unemployment do not refute the rational expectations life-cycle model. Here we checked how expenditure behaves around retirement during uncertain periods.

Our paper has three main objectives. First, we provide causal evidence of the effect of retirement on household expenditure using an instrumental variable framework in two periods: before and after the onslaught of the sovereign debt crisis in Greece. For the sake of exposition, we refer to these as earlier and later periods based on when the first Memorandum of Understanding (MoU) was agreed upon (May 2010). Because the role of spouses in understanding household decisions (Stancanelli & Van Soest, 2012) is central, yet relatively underexplored, we report estimates for both own and spousal retirement effects. Second, we offer evidence that any observed expenditure drops in households where the head is retired are explained by the implementation of pension reductions in the later period. The Greek pension reforms that followed from the debt crisis initially affected certain age groups appreciably more than others, and thus provide a unique opportunity to analyse the role of those retirement income cuts on household consumption. Third, we show that dependency on pension income is central to the decline in household expenditures, conditional on facing declines in pension income. Detailed information on all sources of a household’s income allows us to construct a pension-dependency measure for retirees in order to examine this aspect. Therefore, we provide evidence regarding the variation in consumption declines under different conditions and different types of households.

To achieve these objectives, we used data from the Greek Household Budget Survey, which is a nationally representative household expenditure survey that collects detailed information on household spending using expenditure diaries. The data covered the period from 2008 to 2018, i.e., 2.5 years before and 7.5 years after the MoU was signed. To address the endogeneity of retirement, we exploited retirement legislation in Greece within an instrumental variables framework in which the probability of retiring increases strongly as individuals reach early retirement age. Hence, we used the early retirement eligibility threshold as an instrument for individual retirement status.

Our first-stage results showed that crossing the early retirement age threshold strongly predicts reported retirement status; the probability to retire increases by 21 percentage points after crossing the age and year-specific. Second-stage results showed that household expenditure decreases by 18.5% for retirees in the full period from 2008 to 2018. However, analysing the effects in the earlier and later periods separately shows that the entire observed decrease in retirees’ expenditure can be mainly attributed to the later period, when the MoU was in place. Thus, we do not find robust evidence of a consumption decline at retirement before the MoU. This finding holds regardless of pension level or the household’s dependency on pension income. Moreover, the decline during the MoU period for those with a high dependency on pension income was mostly attributed to reductions in work-related expenses and expenditure on non-necessity goods.

To explain the decline observed in the subsequent, during MoU period, we focused on pension reforms that severely affected retirees’ pension income. In particular, we considered the first and most severe cut in pensions for a subgroup of retirees: those individuals below the age of 60. We found that a large fraction of the decline we observed in expenditure during the later period can be attributed to the pension reduction (approximately 6%) this group faced. The control group (retirees above 60 who did not face such a reduction) showed no decline in expenditures during the same period. Since we are comparing the household expenditure of retirees in this exercise, the treated and control groups did not differ from each other (because both groups are retired), except in exposure to the pension income cut. Our results failed to predict an unexplained consumption drop at retirement during the MoU period once pension cuts were accounted for. In other words, the implemented pension income reductions fully explained the observed expenditure drop at retirement.

We assessed whether dependency on retirement pension matters for the observed decline in spending for those who faced income cuts during the MoU. Bernheim et al. (2001) reported a negative correlation between wealth and a decline in consumption at retirement, as well as smaller declines in retirement consumption for those with higher income replacement rates. Here we split individuals into two groups based on the share of retirement pensions relative to their total household income. We found no effect of retirement on household expenditures for households that did not solely depend on retirement pensions. In contrast, households in which pension income was a significant share of total income showed a significant decline in expenditures during the MoU. This finding implies that households that had other sources of income (or had higher savings or wealth pre-retirement) did not react strongly to reductions in pension incomes and were able to smooth consumption, even in the face of cuts in retirement income.

Our paper contributes to consumption implications of transitions to retirement in several respects. First, we provide a novel assessment of the robustness of the rational expectations life-cycle theory in a hitherto unexamined setting during a particularly turbulent period, i.e., during the sovereign debt crisis in Greece. Extreme conditions -i.e., economic uncertainty and institutional changes- during that period provide a rich opportunity to test the inviolability of well-established, yet empirically debated, theories. Furthermore, understanding the patterns in expenditure around retirement in both good and turbulent times is critical for policy purposes, given the large-scale anticipated demographic changes that will cause transitions to retirement to be more frequent. According to the OECD, Greece has a rapidly aging population, with people over the age of 65 years accounting for more than 20% of the population. Moreover, the old-age dependency ratio, defined as the number of those older than 65 years per 100 working age (20–64) individuals, is expected to reach 40 by 2025, which will be one of the highest among OECD countries (OECD, 2017). At the same time, our findings are also relevant for researchers who examine individual spending decisions and, in turn, have implications for policy. These policy implications are of relevance not only for Greece but for many countries around the world currently faced with the cost-of-living, energy, and public health crises that impact pensioners, and particularly the most vulnerable of them, harder relative to other groups (PLSA, 2022). Analysis on the topic can shed light on mitigating policies that need to be put in place for these groups.

Second, the drop in expenditures at retirement has been tested both spatially and temporally, with early evidence from the US (Bernheim et al., 2001; Hamermesh, 1984); the UK (Banks et al., 1998); Italy (Battistin et al., 2009); and Germany (Schwerdt, 2005), as well as more recent evidence from Spain (Luengo‐Prado & Sevilla, 2013); China (Li et al., 2015); France (Moreau & Stancanelli, 2015); Iceland (Olafsson & Pagel, 2018); and Australia (Atalay et al., 2019). While most studies provide evidence of a retirement-consumption puzzle, Aguiar and Hurst (2005, 2013) and Hurst (2008) cast doubt on its existence. Explanations for the puzzle range from a reduction in work-related expenses, changes in bargaining power (Lundberg et al., 2003; Romm, 2015), and health shocks (Olafsson & Pagel, 2018) to the arrival of adverse information (Banks et al., 1998; Bernheim et al., 2001). In their literature review, Hurd and Rohwedder (2003) argued the necessity of investigating the role of anticipation in the observed drop in consumption. Using subjective retirement expectations data, Haider and Stephens Jr. (2007) reported a drop in expenditures. While they investigated a number of plausible explanations for the drop, they concluded that more work was needed to identify additional causes. We add to this literature by focusing on the role of pension cuts during an unstable period in explaining the expenditure changes at retirement.

Third, we make an important contribution to the literature that analyses the role of individual and household heterogeneity in consumption smoothing behaviour during periods of crisis or income shocks. Fallon and Lucas (2002) summarized the findings of the limited number of studies on household responses to financial crisis and report important differences between rich and poor households in their ability to smooth consumption. Bernheim et al. (2001) examined the drop in expenditure by wealth and income quartiles and reported notable differences between these groups. Similarly, Bloemen and Stancanelli (2005) concluded that allowing for household heterogeneity in terms of wealth is important in detecting discernible differences in food consumption. There is some evidence to suggest that the young and old behave differently during periods of recession (Crossley et al., 2013), yet no previous study has focused specifically on the household expenditure of retirees during these periods. By assessing the drop in expenditures by low- and high-pension-dependency households, we also contribute to this body of work.

The remainder of the paper is structured as follows. Section “Institutional background” outlines the economic background of and institutional changes the Greek pension system has undergone since 2008. Section “Data” presents the data sources and Section “Empirical Strategy” describes the empirical strategy. Section “Results” presents our analytic results, and Section “Conclusions” concludes.

## Institutional Background

The Greek pension system is a social insurance system predominantly funded by employee contributions. For decades it was highly fragmented, with various occupational groups establishing tailor-made pension schemes. Deficits were accumulating, and eventually questions regarding the system’s sustainability were raised (Tinios, 2010). Yet unlike other European countries implementing structural reforms due to pressing demographic and fiscal trends (Casey, 2012), political, social, and institutional inertia in Greece did not allow for similar action to be taken despite the alarming signs. Pressure on the pension system intensified, leading to claims about a strong causal link between the structural imbalances of the pension system and the subsequent debt crisis (Nektarios & Tinios, 2019).

When the global financial crisis hit in 2008, Greece was heavily exposed to international financial pressures and its own economic and fiscal deficiencies. Unemployment reached record levels and the government debt-to-GDP ratio escalated to 146.2% in 2010 (Eurostat, 2018). A rescue plan for economic adjustment and fiscal consolidation was agreed upon in May 2010, leading to the first Memorandum of Understanding (MoU) between the Greek government, the European Commission (EC), the European Central Bank (ECB), and the International Monetary Fund (IMF) (Economou et al., 2017). Two subsequent MoUs were signed in March 2012 and August 2015, which introduced a number of market and institutional reforms and implemented constraints on public expenditure (Maresso et al., 2015).

**Fig. 1:**
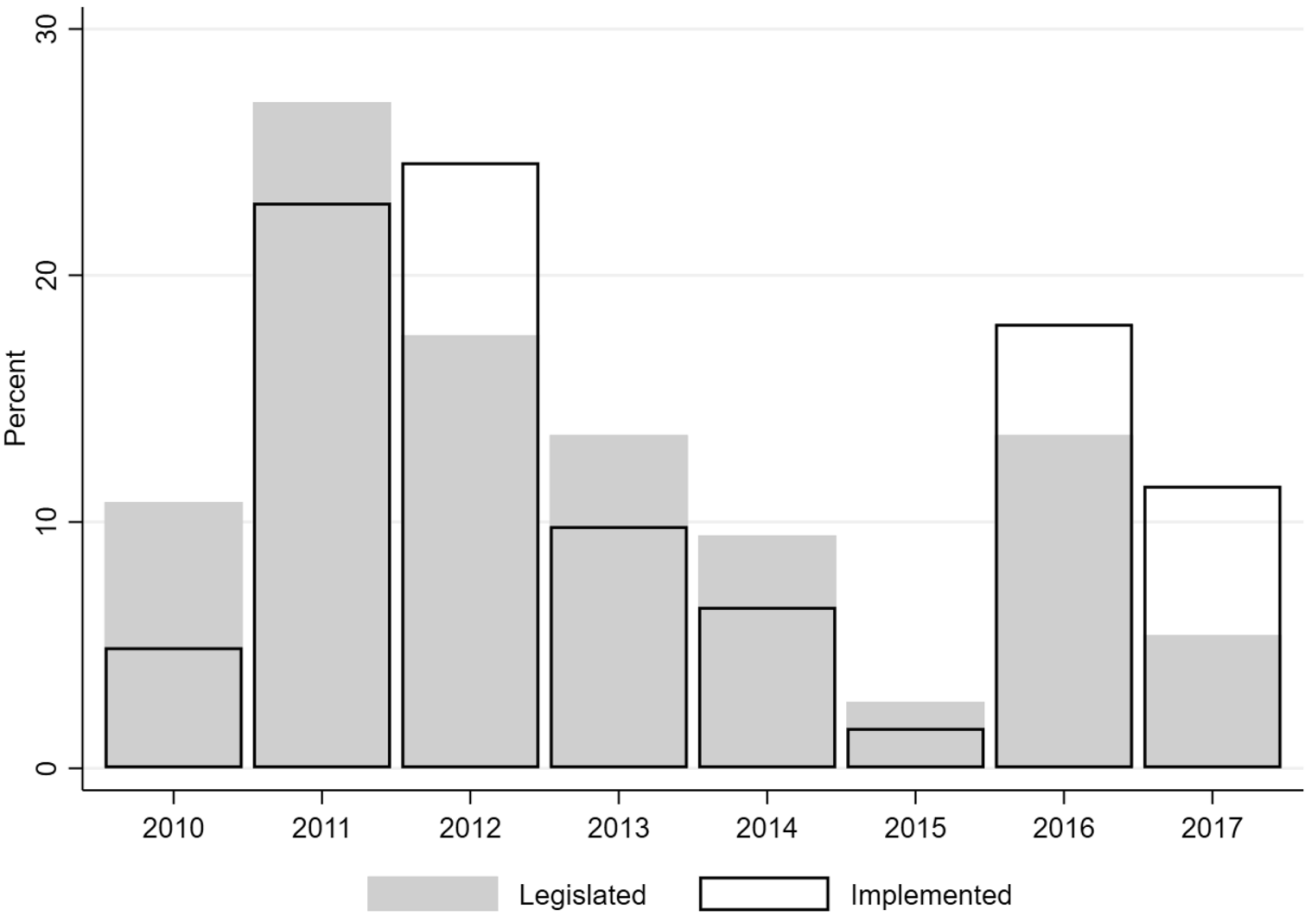
Legislated and implemented reforms related to pensions. *Source:* LABREF database, European Commission. *Notes:* The sample size (all years) is 74 reforms that were relevant to pensions and pensioners

During this period, the pension system underwent a number of reforms. Using information from the European Commission’s LABREF database, Fig. 1 depicts the legislative and implementation intensity of pension-related reforms. The first, which was signed in July 2010 (Act 3863/2010), redefined the pension calculation formula for the younger generation. It also increased the official pension age to 65 years, with an early retirement age set at 60 years, but was quite protective of those about to retire (Panageas & Tinios, 2017). In 2011, various Acts were introduced and, in 2012, the official retirement age was further increased to 67 years for both genders, with the early retirement age reset to 62 years (National Actuarial Authority, 2015; Symeonidis, 2016; Tinios, 2016). Under a socially and politically polarised landscape, new legislation was introduced in May 2016 (Act 4387/2016) that generalised post-2010 changes to the entire population.

Yet what really affected pensioners during this period were the pension cuts. Often the only criterion for the pension cuts was the size of the pensions, without any justification on the basis of age or contribution years (Lyberaki & Tinios, 2016, 2016; Lyberaki et al., 2015; Tinios, 2016). The first cut was applied in May 2010, when Act 3845/2010 abolished the 13th and 14th payments that pensioners had been receiving as Christmas and Easter bonuses; therefore, the cut was effectively implemented in December of that year, when the next Christmas bonus was due. A series of pension cuts targeting young retirees and high pensions were implemented in the second half of 2011. The first wave of cuts was applied to the main pensions of those younger than 60 years old, and ranged from 6% for pensions over €1700 per month to 10% for pensions over €3000. Progressive cuts followed for both high main and high auxiliary pensions.,^1^,^2^ Further reductions in main pensions were implemented later that year, with additional cuts for pensioners younger than 55 years old (40% for pensions higher than €1000) and those 55–60 years old (40% for pensions higher than €1000). The second MoU in March 2012 brought further reductions, which still targeted higher pensions (12% for main pensions higher than €1300 and between 10 and 20% for auxiliary pensions). These were further reduced in 2013 (between 5 and 20% for total pensions over €1000). Bonus (i.e., the 13th and 14th) auxiliary pensions were also abolished that year.

**Fig. 2:**
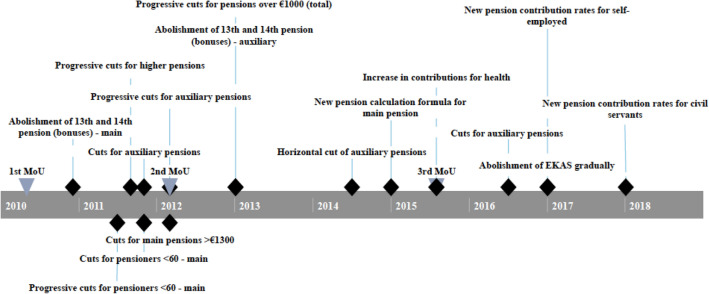
Timeline of implementation of major pension reforms. *Sources:* National Actuarial Authority (2018), European Trade Union Institute (2017), Tinios (2013). *Notes:* Dates refer to implementation rather than legislation of reforms

In July 2014, a first horizontal cut of 5.2% was applied to all auxiliary pensions based on Act 4254/2012. The third MoU, in August 2015, did not introduce further pension cuts, though it increased health insurance contributions to 2% and 5% for main and auxiliary pensions, respectively. It decreed that the Social Solidarity Benefit (*EKAS*) for lower pensions would be abolished by the end of 2019, and introduced new pension contribution rates; however, these did not affect those already receiving a pension. Figure 2 summarises the timeline of major pension reforms of that period.

## Data

Our analysis is based on individual and household-level data drawn from the Greek Household Budget Survey (HBS) provided by the Hellenic Statistical Authority (EL.STAT). The HBS is a national survey that collects information from a representative sample of households regarding their composition, member demographics and employment status, and living conditions; however, the main focus is on household expenditure on goods and services as well as income. The survey runs annually from 2008 onward, and our data cover up to 2018. For each surveyed household, the person perceived by other members as chiefly responsible for decision-making and household management responsibilities is considered to be the household head. For the 2008–2018 waves, the HBS data contain information on 125,652 individuals from 51,840 surveyed households. Although the HBS is a rotating panel, identifiers that would allow to follow individuals and their households over time were not available on data protection grounds. This restricts us from pursuing an analysis that would exploit within household variation over time. Because information on expenditure is reported only at the household level, we restricted our sample only to household heads, i.e., 51,848 observations in total. Moreover, to ensure that we retained married couples in the sample and avoided cohabiting ones, we dropped those who were unmarried or widowed living in a household with more than one member. Moreau and Stancanelli (2015) discussed how cohabitation is more often linked to dual earning, which could potentially affect the results. Even if this could result in some sort of selection bias, there were very few observations in our case, especially in the age brackets around retirement age which were considered in our analysis. We also excluded households in which both the head and the spouse reported “Working” as their main activity status, yet reported that the household’s main income source was unemployment benefits. Finally, we excluded households in which the household head and the spouse were of the same gender, most likely due to misreporting as same-sex marriage had not been legalized in Greece.^3^ These exclusion criteria resulted in losing 5519 observations, or 10.6% of our original sample.

Our dataset included information on a number of subcategories of expenditure: food and nonalcoholic beverages; alcoholic beverages and tobacco; clothing and footwear; housing, water, and electricity; health; household equipment; transport; communication; recreation and culture; restaurants; and miscellaneous goods and services. Although household budget data have been extensively used in related literature (Hurd & Rohwedder, 2008), in our analysis the expenditure categories were strictly separated based on whether a good was durable or on the setting they served (health, labour, etc.).

To analyze the effects of retirement and joint retirement on household expenditures, we constructed a binary indicator for whether heads and their spouses were retired or not. To avoid issues related to reporting bias, we did not use the individual’s perceived status, e.g., a person who was formally retired but identified as working. Instead, retirement status was set equal to 1 if an individual’s main activity was recorded as “Retired” and 0 if it was recorded as “Working.” In this way, we did not include the unemployed, students, those performing domestic tasks, the disabled, and those in military or community service in our estimation samples.

Table 1 presents descriptive statistics on basic variables, for both the total sample and the two groups of retired and non-retired participants. Our sample was restricted to those 15 years on each side of the early retirement age (ERA hereafter) and who had non-missing information on their own and their spouse’s retirement, which left 10,053 observations. ERAs have been defined using the reports of the National Actuarial Authority (National Actuarial Authority, 2012, 2015, 2018). For both genders, the ERA was set to 55 years until 2010, and had been increased to 60 years old by 2012. For the period 2013–2018 it was set to 62 years old. When comparing retirees relative to non-retirees, we observed that they varied in terms of household composition. Non-retirees lived in households of bigger size than retirees (3.4 vs. 2.5 members), mainly due to a larger number of children living with them. Total income was higher for non-retirees than retirees (€38,588 vs €30,427).

**Table 1:** Descriptive statistics on basic variables. *Source*: Household Budget Survey, 2008–2018; Hellenic Statistical Authority (EL.STAT)

	Total sample [1]	Retired [2]	Non-retired [3]	Difference: [3] − [2] [4]
Retired	.494	–	–	–
Spouse retired	.414	.716	.120	− .596***
Age (years)	59.9	66.9	53.0	− 14.0***
Female	.061	.061	.061	.000
Spouse gender	.939	.939	.939	− .000
Primary schooling	.252	.341	.165	− .176***
Secondary schooling	.307	.264	.349	.085***
Tertiary schooling	.301	.240	.360	.120***
Still studying	.074	.045	.102	–
Household size (in persons)	2.9	2.5	3.4	.854***
Number of children in household	.357	.105	.602	.498***
Economically active in household	2.08	2.12	2.03	− .086***
Monetary income (in euros)	30,074.8	26,184.3	33,869.1	7684.8***
Total income (in euros)	34,558.3	30,426.5	38,588.0	8,161.5***
Income source: Self-employment	.200	.056	.341	.285***
Income source: Property income	.006	.007	.006	− .000
Income source: Pensions & retirement benefits	.437	.850	.034	− .816***
Income source: Unemployment benefits	.000	.000	.000	− .000
Income source: Other benefits	.003	.002	.004	.002*
Household expenditure variables (in euros)				
Total expenditure	29,671.3	23,940.0	35,269.7	11,338.9***
Food & non-alcoholic beverages	5016.7	4532.5	5489.1	956.6***
Work-related expenses	4874.0	3500.3	6213.8	2713.6***
Necessity expenses	16,204.0	14,471.6	17,893.6	3422***
Non-necessity expenses	16,616.5	7738.5	13,423.3	5684.8***

Column 4 reports mean differences *t*-tests results

^†^p < .1. *p < .05. **p < .01. ***p < . 001

The lower panel of Table 1 presents descriptive statistics regarding household expenditure for the total period. The average total expenditure in households in which heads were not retired is €11,339 higher than their retired counterparts. The higher expenditure differences between the two household types were observed for transport, housing, water, electricity and fuels, and restaurants and hotels. To examine whether reductions in consumption were associated with specific activities, we grouped the disaggregated expenditure categories into four broad ones (Aguiar & Hurst, 2005, 2013; Battistin et al., 2009; Blundell et al., 2016; Lundberg et al., 2003). These were food-related expenses (excluding alcohol and tobacco); work-related expenses (transport and clothing expenses); necessity expenses (food, housing, water, electricity, health, and clothing); and non-necessity expenses (alcohol, tobacco, household equipment, transport, communication, recreation, and hotels and restaurants). Mean differences were all statistically significant at the 1% level (column 4).

## Empirical Strategy

### Model Specifications

This section outlines our empirical framework. We tested how household expenditure changes at retirement conditional on spouse’s retirement status, i.e., we were interested in the effect of a couple’s retirement on household expenditure. Prior evidence has demonstrated the importance of spousal retirement not only in spending behavior but also in own retirement decisions. In particular, Stancanelli and Van Soest (2012), Lundberg et al. (2003), and, more recently, Moreau and Stancanelli (2015) have demonstrated this in the context of household expenditures, while Whitaker and Bokemeier (2018) and De Preter et al. (2015) have shown this interdependence when considering the timing and incidence of own retirement decisions. Our empirical model is therefore similar in spirit to that of Moreau and Stancanelli (2015). In its simplest form, our main specification is as follows:

1 $${Y}_{ht}= {\alpha }_{0}+ {\beta }_{1}{R}_{mht}+ {\beta }_{2}{R}_{fht}+f(Ag{e}_{mht})+ f(Ag{e}_{fht}) +{\delta }_{t}+ {\varepsilon }_{ht}$$

where ${Y}_{ht}$ represents total household expenditure of household *h* at time *t*. We are interested in ${\beta }_{1}$ and ${\beta }_{2}$, which represent the coefficients of interest for both male (*m*) and female (*f*) partners in household *h* at time *t*. We also control for a second-order polynomial in age of both partners, year dummies to account for common exogenous shocks, regional fixed effects to capture permanent differences, and household-level control variables, such as household size and number of dependent children, so that our estimates are net of household composition effects. Last, ${\varepsilon }_{ht}$ is the disturbance term.

While ${\beta }_{1}$ and ${\beta }_{2}$ in Eq. (1) provide estimates of the effect of retirement, they are clearly biased since retirement is an endogenous choice; individuals can choose to retire earlier or later depending on factors such as health and wealth. Ideally, to overcome this identification problem retirement status should be randomly assigned across individuals in our data. However, this is infeasible. In the absence of such a randomized experiment, we exploit Greek legislation regarding early retirement to uncover causality. Exploiting the fact that the probability of being retired increases strongly as individuals reach ERA, we use an instrumental variables approach. The necessary variation required for identification comes from the exogenously set ERAs in Greece, ERA threshold that are set separately for males and females, and the fact that those ERAs were increased twice during the period under consideration, in 2010 and in 2013.

We apply two-stage least squares (2SLS) and instrument the retirement status of both partners in the first stage with a binary instrumental variable equal to 1 if the individual has crossed the ERA and 0 otherwise. Whenever interaction terms between retirement status and centered age are included in the empirical specifications, they are also instrumented with interactions between dummy variables about crossing the ERA and centered age. Therefore, we estimate first-stage regressions of the form

2 $${R}_{mht}= \gamma + {\gamma }_{1}{Z}_{mht}+{{\gamma }_{2}Z}_{fht}+f(Ag{e}_{mht})+f(Ag{e}_{fht})+{\delta }_{t}+ {\nu }_{ht}$$

and

3 $${R}_{fht}= \tau + {\tau }_{1}{Z}_{fht}+{{\tau }_{2}Z}_{mht}+f(Ag{e}_{fht})+f(Ag{e}_{mht})+{\delta }_{t}+ {u}_{ht}$$

where ${R}_{mht}$ and ${R}_{fht}$ represent retirement status (1 = retired; 0 = active in the labour market) of the male and female partner in household *h* at time *t*, respectively. $f(Ag{e}_{mht})$ and $f(Ag{e}_{fht})$ are second-order polynomials in the age of male and female partners in order to account for nonlinear lifetime expenditure profiles. Year fixed effects are denoted by ${\delta }_{t}$, and ${\nu }_{ht}$ and ${u}_{ht}$ are the respective error terms of both equations. ${Z}_{mht}$ and ${Z}_{fht}$ are binary instrumental variables defined as follows:

$${Z}_{mht}=1 if\, Ag{e}_{mht}\ge ER{A}_{mt};\,0 \quad if\, Ag{e}_{mht}<ER{A}_{mt}$$

$${Z}_{fht}=1 if\, Ag{e}_{fht}\ge ER{A}_{ft};\,0\quad if\, Ag{e}_{fht}<ER{A}_{ft}$$

As is standard in the IV literature, we interpret the coefficients of retirement status indicators in Eq. (1) as the local average treatment effects (LATE) of ERA eligibility on expenditure behaviour. In other words, we estimate average treatment effects for those who exit the labour market into retirement given their eligibility, i.e., the ERA compliers. However, because the total period under study is not homogeneous, comprising a stable (before the MoU period) and a turbulent (during the MoU) sub-period, the LATE could not be the same in the two periods. This is because the set of compliers will differ given the different aggregate environment as well as other observable characteristics, such as age. An instrumented difference-in-differences approach would allow to directly test for such changes in consumption at retirement before and during the MoU period. However, as previously mentioned, longitudinal identifiers of households and their members were not available. Hence, an approach relying on within-unit variation over time was not possible to be pursued. Therefore, we estimate Eq. (1) for the total period, as well as by sub-period, i.e. before and during the MoU, relying on cross-sectional variation. For a more detailed examination of how the impact of retirement on expenditure evolves throughout the period under study, we include interactions of retirement with year dummies in alternative specifications, as follows:

4 $$\begin{gathered} {Y}_{ht}= {\alpha }_{0}+ {\beta }_{1}{R}_{mht}+ {\beta }_{2}{R}_{fht}+{\beta }_{3}{R}_{mht}\times {\delta }_{t}+{\beta }_{4}{R}_{fht}\times {\delta }_{t} \hfill \\ \quad\quad\,\,+\,f(Ag{e}_{mht})+ f(Ag{e}_{fht}) +\,{\delta }_{t}+ {\varepsilon }_{ht} \hfill \\ \end{gathered}$$

For robustness, we use samples that cover varying time windows around ERA, and estimate placebo regressions using fake ERAs that range in time before the actual ERA.

### Instrument Relevance and Validity

A good instrument must fulfil two criteria: relevance and validity. Validity requires that the instrument is correlated with household expenditure only through retirement. While validity cannot be formally tested, given that the ERAs are exogenously set by the government, it is plausible that crossing the ERA threshold is related to household expenditure only through transitions from being labour-market active to retirement. Nevertheless, we conducted placebo tests reported later to support instrument validity. Relevance, on the other hand, refers to a strong first-stage relationship between the instrument and the endogenous variable. In other words, relevance requires that observed retirement statuses for household heads and their spouses are strongly predicted by the instruments ${Z}_{mht}$ and ${Z}_{fht}$, respectively. We present some descriptive evidence regarding the relevance of ERA as an instrument, and then discuss the first-stage results.

**Fig. 3:**
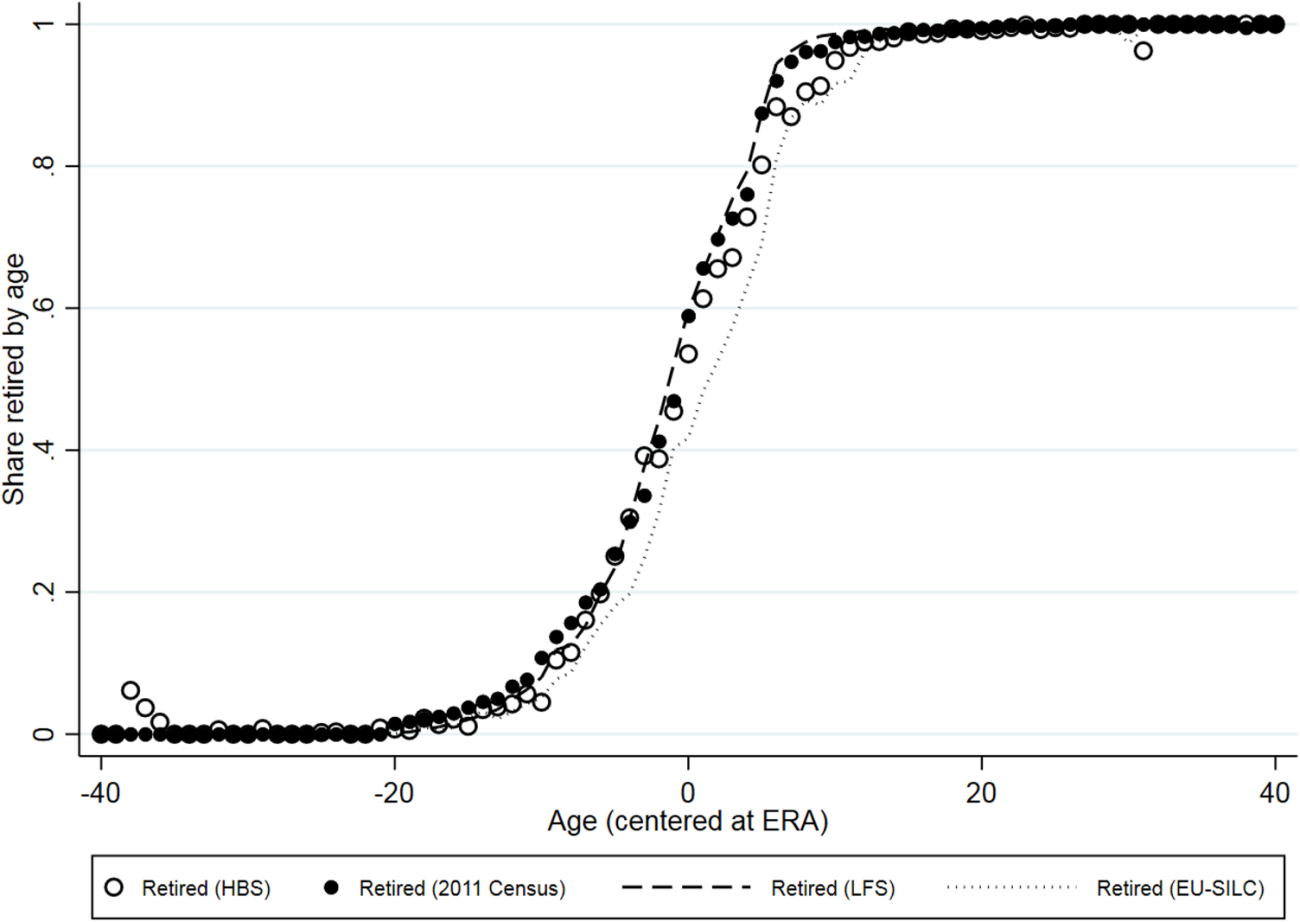
Retirement shares by age. *Source:* Household Budget Survey (2008–2018); Greek Census (2011); Labour Force Survey (2015–2018); EU-SILC (2009–2017); Hellenic Statistical Authority (EL.STAT). *Notes:* Shares are weighted with each survey’s respective weights. ERAs are specific to the survey's time period

We begin by a graphical observation of retirement behavior around ERA. Figure 3 scatters the share of those retired by age, where age has been centered at year and gender-specific ERAs, using HBS data. It also graphs the age-specific shares of retirees using other available databases to verify the representativeness of our HBS sample. More specifically, we drew data from the 2011 Census, the Survey on Income and Living Conditions (EU-SILC) for 2009–2017, and the Labour Force Survey (LFS) for 2015–2018.^4^ Similar to the HBS data, we classified as non-retired those either employed or unemployed. Hence retirees were compared with individuals who were active in the labour market. First, the graph shows that the HBS sample is representative compared with other datasets. Retiree shares are nearly identical when benchmarked to those obtained using Census, LFS and EU-SILC data. Second, it suggests that although there are people who tend to retire before reaching ERA, the majority retire right on or after crossing this threshold.

**Fig. 4:**
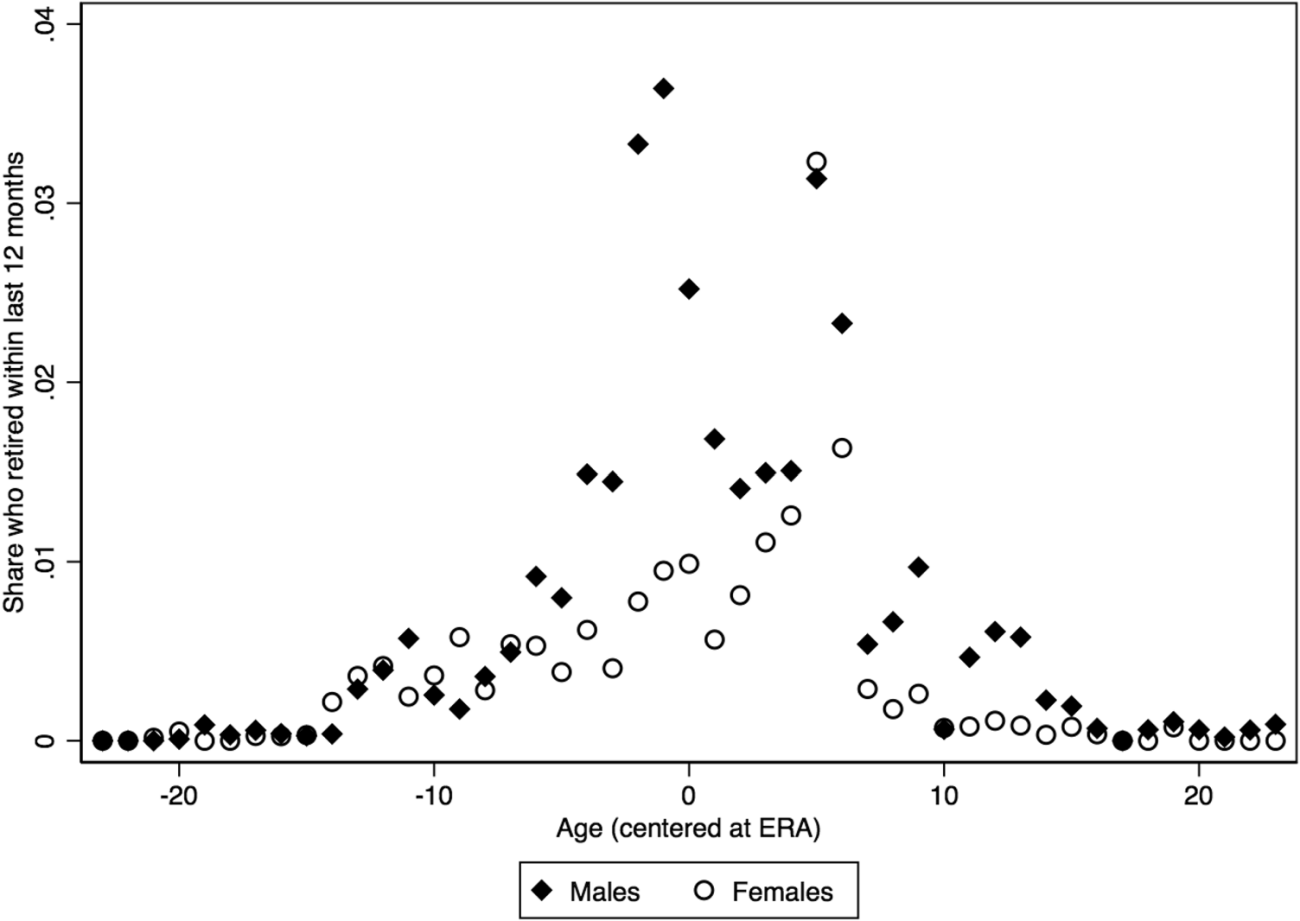
Transitions to retirement by gender. *Source:* Labour Force Survey (2015–2018); Hellenic Statistical Authority (EL.STAT). *Notes:* Shares are weighted by the sampling weights

**Fig. 5:**
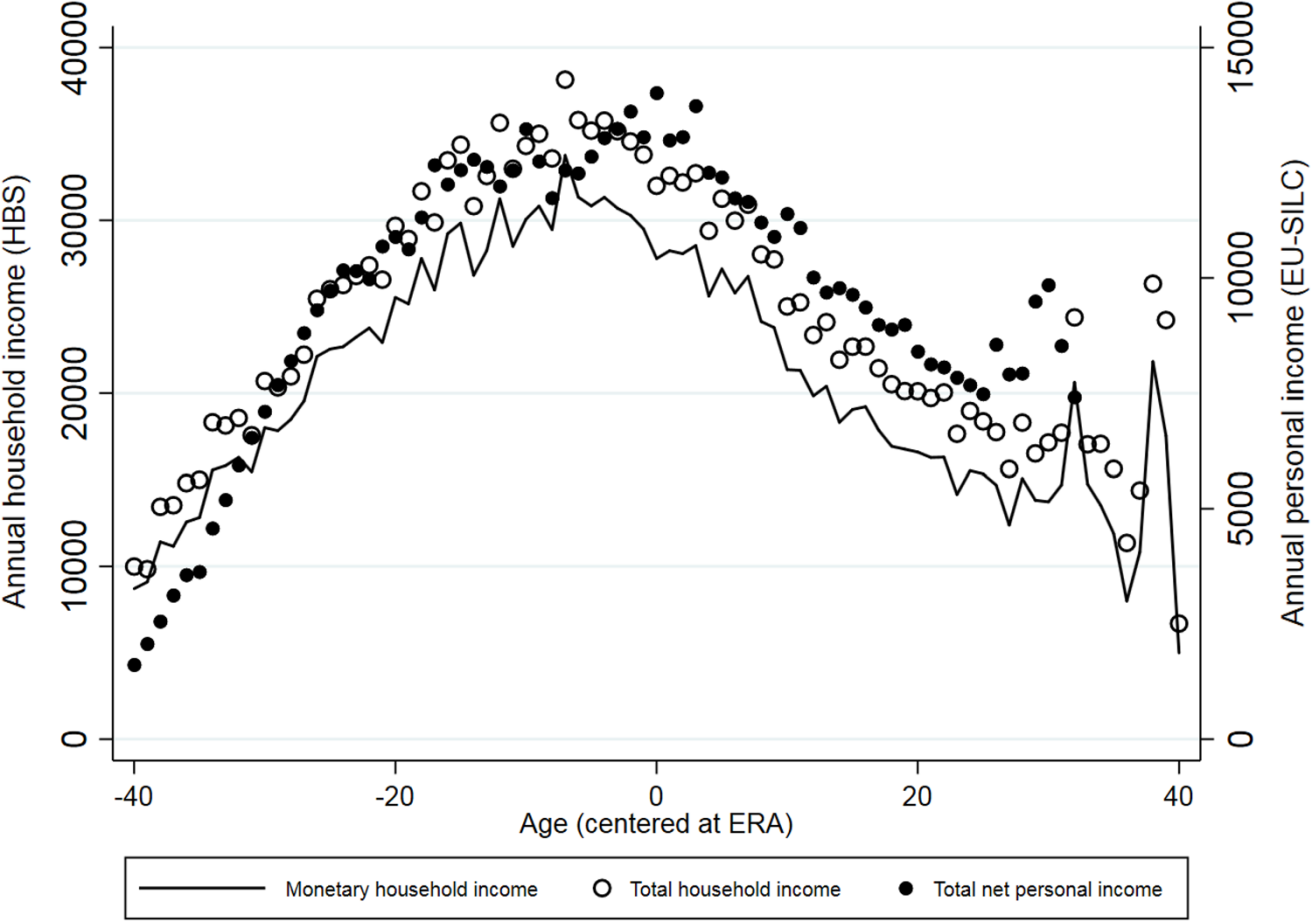
Income levels around retirement. *Source:* Household Budget Survey (2008–2018); EU-SILC (2009–2017); Hellenic Statistical Authority (EL.STAT)

To address any concerns regarding the issue of retiring before reaching the ERA, we used the LFS data to construct age- and gender-specific shares of those who retired in the last 12 months, conditional on being active in the labour market one year before they were surveyed. Figure 4 displays the results. There was a discrete jump at the cutoff age, especially for males, with a relatively low incidence of people retiring considerably before reaching the ERA.^5^ Moreover, it seems that relative to males, females tended to retire later. Figure 5 plots monetary and total household income and total net personal income around the ERA. Total household and personal incomes declined after ERA, although monetary household income started declining a few years before. This income loss observed around the ERA was expected to affect household expenditure behavior, since heads and their spouses were on the margin of early retirement.

**Fig. 6:**
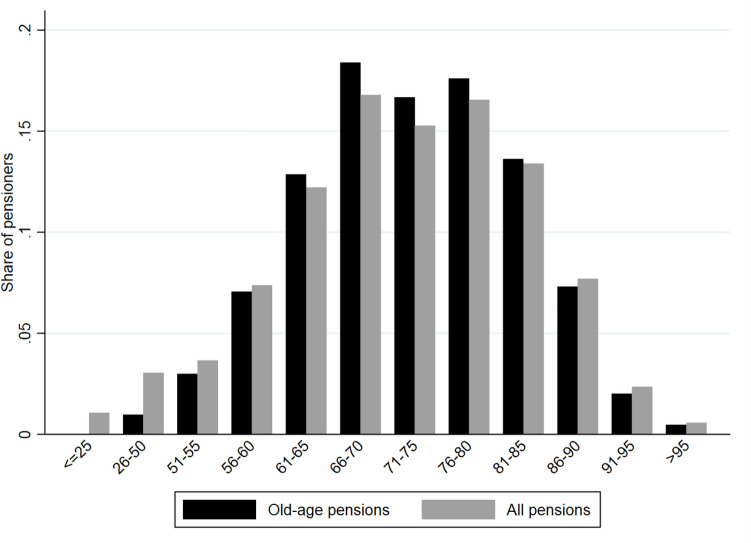
Pensions by type and age group. *Source:* Ministry of Labour and Social Affairs (MoLSA). *Notes:* The data cover the period October 2013-December 2016. All pensions include old-age, disability, death, and other pension types. Age groups are the default ones as reported in the source

To provide further graphical evidence on the timing of transitions to retirement in Greece, we used publicly available information provided by the Ministry of Labour and Social Affairs (MoLSA). From October 2013 onward, they have been uploading online monthly reports on pensions by type (old-age, disability, death, other), age group, and fund. Statistics on those reports are based on administrative information on the universe of pensioners collected through “Helios,” a Unified Pension Monitoring and Payment System that was developed under the provisions of Act 4093/2012. Scattered data from information systems belonging to 92 different pension funds were combined for the first time and identify about 2.7 million pensioners. We used online data (2013M10-2016M12) to plot the shares of all pensioners and old-age pensioners by age group (Fig. 6). Although information by exact age or narrower age groups was not available online, it seems that the share of those receiving old-age pensions jumped discontinuously for the 56–60 age group and this was more apparent for the 61–65 age group. Moreover, it seems that the pension types being claimed before reaching ERA were not age-related; for instance, based on disability.

## Results

### First-Stage Results

Using various data sources, we found that ERA was indeed a relevant instrument for retirement status. To demonstrate the strength of the instrument, Table 2 presents first-stage regression results for three samples: the total period (2008M1–2018M12); the pre-MoU period (2008M1–2010M5); and the period during the MoU (2010M6–2018M12). The probability of retirement after crossing ERA increased by 21.2% for household heads (column 1) when considering the total period. The instrument was particularly strong in more recent years, i.e., during the MoU period (panel C). Regarding household heads, the effect of crossing the ERA threshold was much weaker in the pre-MoU period, relative to the period during the MoU, which reflects the fact that more people tended to retire early.

**Table 2:** First-stage results. *Source:* Household Budget Survey, 2008–2018; Hellenic Statistical Authority (EL.STAT)

Dependent variable:	Own retirement	Spousal retirement
	[1]	[2]
Panel A: Total period (2008M1–2018M12)		
Own age > ERA	.212*** (.018)	− .026 (.017)
Spouse age > ERA	.036* (.016)	.136*** (.019)
Household size (persons)	− .014** (.005)	− .016*** (.005)
Dependent children in household	− .041** (.013)	.035** (.012)
F-test of excluded instruments	57.18	40.04
Observations	10,053	10,053
Panel B: Before MoU (2008M1–2010M5)		
Own age > ERA	.092** (.032)	.012 (.032)
Spouse age > ERA	.078† (.045)	.134** (.044)
Household size (persons)	− .020* (.010)	− .011 (.008)
Dependent children in household	− .038 (.025)	.032 (.024)
F-test of excluded instruments	20.26	4.60
Observations	1771	1771
Panel C: During MoU (2010M6–2018M12)		
Own age > ERA	.234*** (.020)	− .037† (.019)
Spouse age > ERA	.025 (.017)	.136*** (.021)
Household size (persons)	− .010† (.005)	− .016** (.005)
Dependent children in household	− .032* (.014)	.038** (.014)
F-test of excluded instruments	57.65	48.89
Observations	8282	8282
Individual controls	Yes	Yes
Household controls	Yes	Yes
Year & region fixed effects	Yes	Yes

Linear probability model estimates using own and spousal retirement as dependent variables. The instrument used is a binary indicator for whether own (spouse) age is greater than the early retirement age (ERA). Controls include a second-order polynomial in age, age-treatment interactions, total household income, household size, and whether dependent children live in the household. Robust standard errors in parentheses

^†^p < .1. *p < .05. **p < .01. ***p < . 001

Regarding spousal retirement (column 2), the probability increased by 13–14% in every period considered. When considering complementarities in retirement decisions, a spouse’s crossing the ERA had a positive impact on household head’s retirement probability when examining the total period (panel A). This effect was weak before the MoU (panel B) and disappeared in the MoU period (panel C). In contrast, household head’s crossing the ERA had no impact on spousal retirement (panels A and B), while it exerted a negative but weak impact during the MoU period. This is consistent with the fact that spouses tended to retire relatively later, as shown in Fig. 4. It is also consistent with prior literature that argues that spouse’s labour supply decisions depend on the head’s income, although the opposite is not necessarily true (Hurd, 1990). In any case, the instrument relevance condition is clearly satisfied, since the instruments were strong predictors of retirement status and the first-stage *F*-statistics were quite high. With respect to other household characteristics, the probability of the household head’s retirement was negatively related to the number of persons and the presence of dependent children in the household in every period. On the other hand, dependent children in the household increased the probability of female retirement.

As outlined in Section “Instrument relevance and validity”, we estimated local average treatment effects (LATE) of ERA eligibility on expenditure behaviour, i.e., we estimated average treatment effects for those who exit the labour market into retirement given their eligibility (the ERA compliers). In our analysis the group of compliers was substantial in size (the size of the complier group equalled the first-stage effect i.e., 0.21). Furthermore, we explored complier characteristics using the ratio of the first-stage effect conditional on a specific characteristic relative to the overall first-stage (Angrist & Pischke, 2008). We observed that compliers were less likely to have children living in the same house. Compliers were also less likely to have higher monetary income, and to have any economically active members in the household. The same patterns held when examining complier characteristics before and during the MoU period.

### Impact of Retirement on Expenditure

Having established (a) a strong first-stage relationship between retirement status and early retirement eligibility for household heads and their spouses, and (b) evidence on complementarities in retirement decisions, we now examine the impact of retirement on household expenditure. Table 3 reports our baseline 2SLS results regarding the impact of own and spousal retirement on total household expenditure. We report results using a simple and an interacted model in Eqs. (1) and (4), respectively. This will reveal whether the overall effects of retirement vary over time.

**Table 3:** Retirement and total expenditure. *Source:* Household Budget Survey (2008–2018); Hellenic Statistical Authority (EL.STAT)

	Total period (2008M1–2018M12)	Before MoU (2008M1–2010M5)	During MoU (2010M6–2018M12)
	[1]	[2]	[3]
Retired	− .204* (.089)	− .142 (.235)	− .178** (.087)
Retired × Age	− .015 (.012)	− .005 (.027)	− .017 (.012)
Spouse retired	− .023 (.130)	.241 (.431)	− .063 (.118)
Spouse retired × Age	− .020** (.006)	− .015 (.016)	− .022** (.007)
Household size (persons)	.129*** (.007)	.137*** (.015)	.128*** (.007)
Dependent children in household	.092*** (.018)	.094* (.045)	.094*** (.019)
Observations	10,053	1771	8282

2SLS estimates. Robust standard errors in parentheses. All models include individual and household controls and region and year fixed effects

^†^p < .1. *p < .05. **p < .01. ***p < . 001

According to the results in column 1, the associated coefficient of household head retirement is equal to − 0.204 and statistically significant at the 5% level.^6^ This implies that household head retirement decreased total expenditure by around 18.5%.^7^ However, an issue with this estimate is that it referred to a period that cannot be considered as homogeneous. While the period before the MoU was rather stable without reforms or large aggregate income shocks, the period during MoU clearly departed from being characterized as normal. Therefore, in columns 2 and 3 we report results for the periods before and during the MoU, respectively. In the pre-MoU period (column 2), no statistically significant effect is observed. Although not statistically significant, this result can be considered as comparable to existing work on the retirement-consumption puzzle with idiosyncratic but no large, negative aggregate shocks. During the MoU period, the coefficient of household head retirement is − 0.178 and significant at the 5% level (column 3). Hence, household head retirement during the MoU period decreased total expenditure by 16.3%.^8^ However, we avoid considering this result based on data from a turbulent period as a test for the retirement-consumption puzzle. The occurrence of large, negative income shocks should generate a consumption decline even for agents in a canonical life-cycle model. Spousal retirement did not have a statistically significant impact on household expenditure. Moreover, the interaction term between spousal retirement and spousal age suggested that consumption decreases by around 2% per year as the spouse gets older. On the other hand, household size and the presence of dependent children in the household increase expenditures significantly and with a similar magnitude before and during the MoU period.

**Fig. 7:**
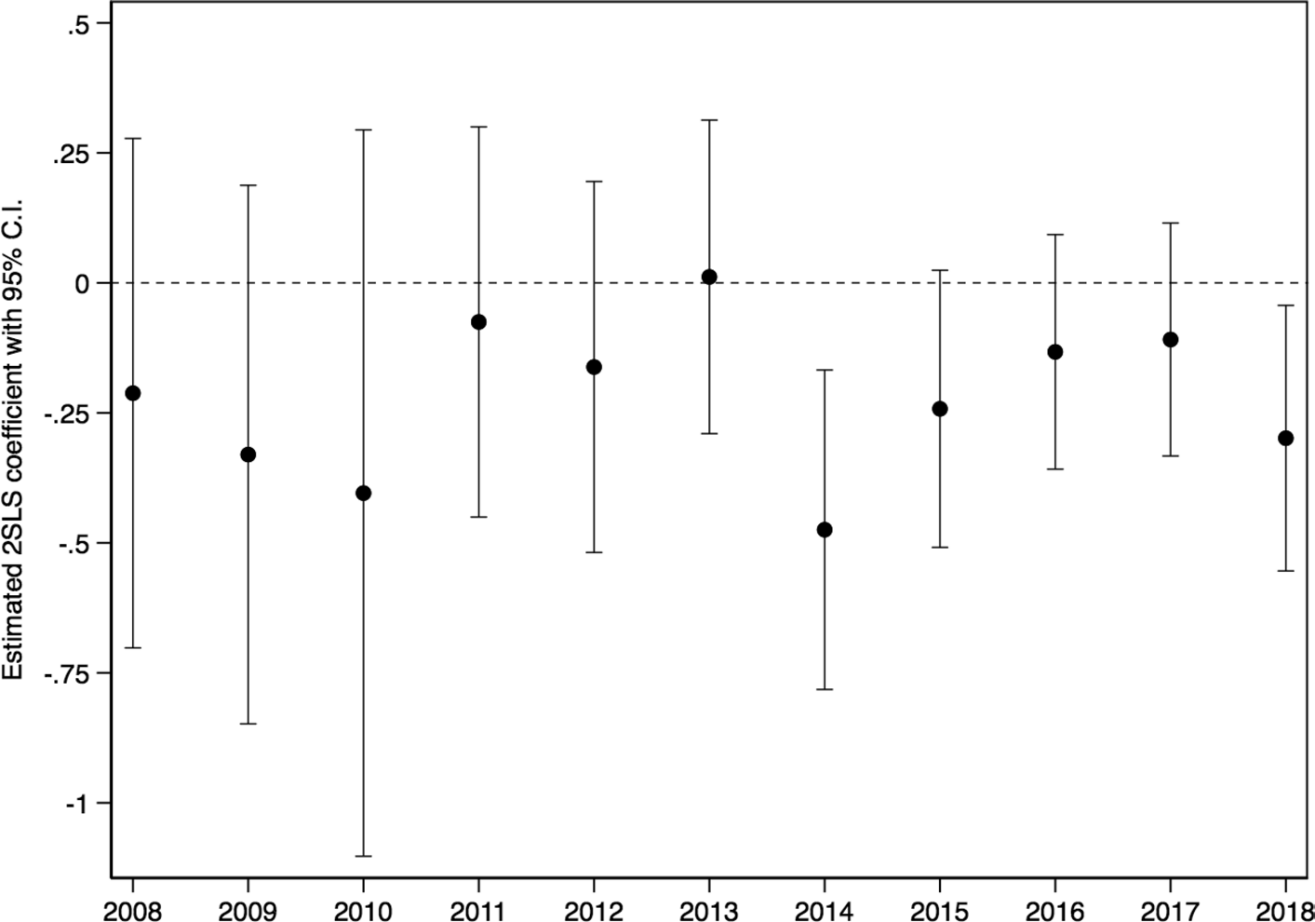
Retirement and expenditure by year. *Source:* Household Budget Survey (2008–2018); Hellenic Statistical Authority (EL.STAT). *Notes:* 2SLS estimates. 95% confidence intervals are based on robust standard errors. All parameters are obtained from models that include individual and household controls and year fixed effects

Figure 7 plots the respective results for household heads, for which we obtain significant estimates, along with their 95% confidence intervals. As in Eq. (4), retirement status has been interacted with year dummies. Austerity measures were first announced in 2010, and in 2014 a 5.2% horizontal cut across all auxiliary pensions was applied. Our year-specific results uncovered a statistically significant expenditure drop at retirement only after 2014, when pension cuts were generalized. Earlier pension reforms and cuts were not associated with significant expenditure decreases. This should be due to the fact that pension cuts in the early MoU years targeted young pensioners and those receiving high pensions, who represented a small fraction of all retirees. Using HBS data, we located groups of retirees who seemed mostly relevant with the interventions displayed in the timeline of Fig. 2, and calculated their share of the total sample of retirees up to 3 months before each intervention. For example, pensioners younger than 60 years old and receiving more than €1700 per month represented only 0.79% of all retirees before July 2011; in addition, cuts in that group were progressive. Similarly, progressive cuts that were applied to those receiving high main and auxiliary pensions (≥ €1400 and ≥ €300, respectively) were relevant for about 17% of our 3-month sample before September 2011. Also, pensioners less than 60 years old and 55 years old who received main pensions over €1200 and €1000 faced a cut of 20% and 40%, respectively, in November 2011. However, both groups represented only 3.3% of all retirees in our data before that measure was applied. Therefore, the negative but noisy 2SLS estimates for the early years in Fig. 7 can be attributed to the fact that initial pension cuts were highly targeted.

### Robustness Checks

#### Placebo Tests

Placebo tests confirm the robustness of our baseline results on total expenditure. Table 4 reports results obtained after estimating Eq. (1) but replacing the actual ERA (column 5) with fake ones ranging 1–4 years earlier (columns 1–4). In this way, we tested whether (a) results were biased by the anticipation effects of retirement on household expenditure behaviour and (b) the identification strategy was threatened by the fact that people in Greece might make systematic use of exception rules in order to claim pensions even sooner than their official ERA. Column 5 reproduces the results from column 1 of Table 4, in which the actual ERA threshold was used. With respect to columns 1 to 4, we see that own retirement status is not significant when using fake ERAs, and the associated coefficients tend to zero and become noisier as we move away from the actual eligibility threshold. These placebo tests provide reassurance regarding any threats imposed by the fact that some people in Greece retire before the actual ERA. To further examine this issue, we looked at individual-level data from the Survey on Transition from Work to Retirement, which is an ad hoc module of the Labour Force Survey. In 2012, LFS respondents aged 50–69 years old were interviewed in order to gather information regarding their transition from the labour market to retirement and the motivations behind it. From the sample of individuals who were allowed to retire early, 76% retired only after they reached 60 years old.^9^ This is the ERA threshold that we used for respondents in the 2012 HBS wave following the recommendations made in OECD and National Actuarial Authority reports. Therefore, our results do not seem to be seriously threatened by people who claim pensions before they reach the ERA threshold.

**Table 4:** Impact of retirement on expenditure: falsification tests. *Source:* Household Budget Survey (2008–2018); Hellenic Statistical Authority (EL.STAT)

ERA specified at	t-4	t-3	t-2	t-1	t = Actual ERA
	[1]	[2]	[3]	[4]	[5]
Retired	− .059 (.146)	− .103 (.137)	− .140 (.130)	− .185 (.144)	− .204* (.089)
Retired × Age	− .027 (.021)	− .027 (.021)	− .022 (.018)	− .035 (.172)	− .015 (.012)
Spouse retired	.103 (.145)	.149 (.158)	.074 (.165)	.066 (.101)	− .023 (.130)
Spouse retired × Age	− .019** (.007)	− .019** (.007)	− .020** (.007)	− .022** (.007)	− .020** (.006)
Observations	10,053	10,053	10,053	10,053	10,053

2SLS estimates. Robust standard errors in parentheses. All models include individual and household controls and region and year fixed effects

^†^p < .1. *p < .05. **p < .01. ***p < . 001

#### Varying Window Width Around the ERA

**Fig. 8:**
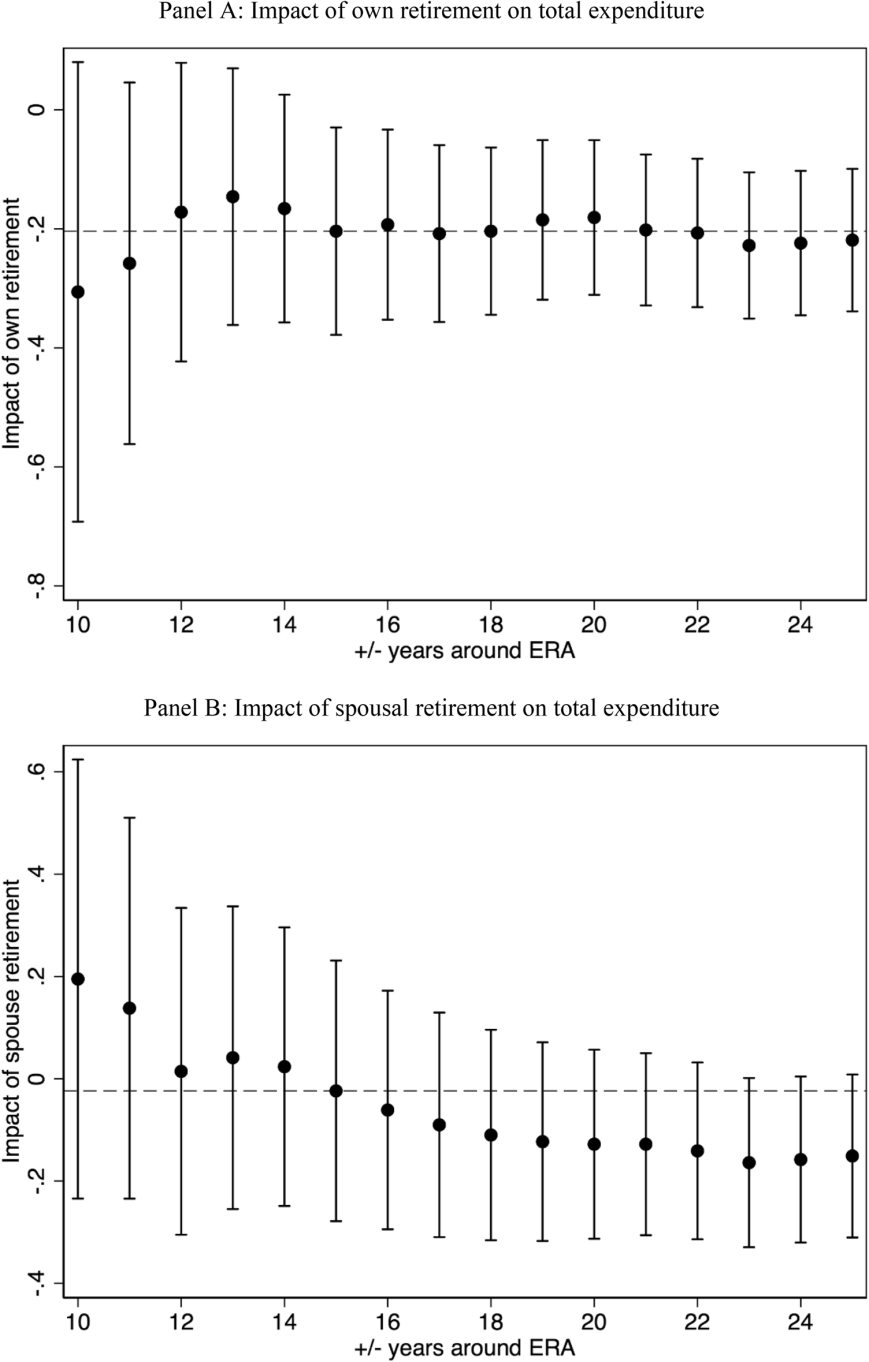
Retirement and expenditure using alternative time windows around the ERA. *Source:* Household Budget Survey (2008–2018); Hellenic Statistical Authority (EL.STAT). *Notes:* 2SLS coefficients with 95% confidence intervals based on robust standard errors. Dashed horizontal lines represent the average baseline effects obtained when estimating the model using a 15-year bandwidth around the ERA. All models control for individual and household characteristics and year fixed effects

Model specifications have been estimated using a bandwidth of 15 years around both sides of the ERA cutoff. We re-estimated the baseline model using alternative bandwidths to check the sensitivity of our 2SLS coefficients. For each alternative bandwidth, Fig. 8 reports the 2SLS estimated coefficients along with their 95% confidence intervals. Dashed lines represent the baseline effect when models are conditioned on the usual 15-year bandwidth and correspond to those reported in Table 3. Regarding the impact of own retirement (Panel A), this is − 0.204 and statistically significant at the 5% level. This result was robust to both wider and narrower bandwidths around the ERA. Using bandwidths that were narrower than 10 years around the cutoff still returned larger coefficients, in absolute terms, but they were not precisely estimated due to considerably smaller sample sizes. With respect to the impact of spousal retirement on total expenditure, the estimated parameters were small and not statistically different from zero (Panel B). Regardless of bandwidth choice, the 95% confidence intervals always crossed zero; this provided further evidence against the existence of a link between spousal retirement and total expenditure.

#### Household Composition

One concern could be that our results were driven by compositional changes within the household. Although our models do control for household composition, we empirically tested for links between household composition and the retirement-consumption puzzle. Table 5 presents the results. Column 1 shows that conditional on income, retirement is associated with a 28% drop in total expenditure. In column 2 we control for household size, measured as the number of people in the household, and a binary variable that indicate whether the household consists of adults only or adults with dependent children. These are the results that have been reported elsewhere in the paper. Controlling for household composition explained part of the drop in consumption, given the total income. Household composition controls had the expected sign, suggesting that expenditure increases with the number of people and the presence of dependent children in the household. An association between household consumption and household size was found in previous studies (Battistin et al., 2009). However, as the unemployment rate increased dramatically during the period covered by our data, there might be changes in household composition because of the crisis; for instance, children moving back in with parents due to unemployment, job loss, or lower pay. Also, it could be the case that adult children chose not to move out of their parents’ household because of financial constraints. Our data are cross-sectional, and hence we cannot observe someone losing their job and moving back to their parents’ household. However, we constructed a variable to indicate the presence of unemployed children in the household and include it as a control in column 3. The results suggest that there is a negative effect of unemployed children in the household, which explained a small part of the drop in post-retirement consumption. Controlling additionally for a variable that indicated whether there were adult children in the household in column 5 did not seem to explain much of the retirement-consumption relationship. The original household composition variables seemed to capture all of the composition-related effects, while adult and unemployed children had a positive and negative relationship to total expenditure, respectively.

**Table 5:** Retirement and total expenditure: differences in household composition. *Source:* Household Budget Survey (2008–2018); Hellenic Statistical Authority (EL.STAT)

	[1]	[2]	[3]	[4]
Retired	− .279** (.093)	− .204* (.089)	− .219* (.089)	− .193* (.090)
Spouse retired	− .091 (.136)	− .024 (.130)	− .025 (.130)	.007 (.132)
Household size	–	.129*** (.007)	.139** (.007)	.121*** (.008)
Presence of dependent children	–	.092** (.018)	.085** (.018)	.096** (.018)
Unemployed children in household	–	–	− .091** (.016)	− .120** (.018)
Adult children in household	–	–	–	.064** (.017)

2SLS estimates. Robust standard errors in parentheses. All models include individual and household controls and region and year fixed effects

^†^p < .1. *p < .05. **p < .01. ***p < . 001

Apart from household composition, household expenditures can be affected by the level of education since the latter is considered a proxy for access to economic resources (Battistin et al., 2009). Normally, the level of educational attainment is determined considerably before eligibility for early retirement; therefore, there should not be significant differences for those at the margins of the ERA cut-off. Otherwise, the observed drop in consumption might be driven by the endogenous sorting of individuals into retirement that is related to their educational decisions. For example, low-educated individuals might retire earlier because they are more likely to sort themselves into low-paying and health-hazardous occupations or because labour market opportunities are scarcer for them, especially in high-unemployment times. On the other hand, high-educated individuals might choose to stay longer in the labour market due to higher returns or better working environments. Hence, we tested whether there were significant differences in educational attainment between retirees and non-retirees. To do this, we used a variable containing the broad level of education for each individual, and found that the main results are robust to the inclusion of education controls.^10^

#### Variation in Consumption Declines

Robust to several tests, our results so far point to consumption declines at retirement during a period when pension reforms and cuts were implemented. This should be expected given that the large, negative income shocks observed during that period could generate such declines; even for agents in a canonical life-cycle model. In this case, it is interesting to examine how these declines vary based on some observable characteristics of the sample. Therefore, we tested whether the changes at retirement observed during the MoU were driven by those retirees who were mostly dependent on their pension income -with no property or other income sources within the household- during bad times. Using the detailed income information in the HBS data, we calculated how dependent a household’s total income is on the head’s pension by dividing total pension income by total household income. This pension dependency ratio ranged between 0 and 1, and based on its distribution we classified households as follows: those with a relatively low dependency on pension income (dependency ratio < 0.68), and those with a high dependency on pension income (dependency ratio ≥ 0.68). Then we ran 2SLS regressions for various periods and report the results in Table 6. We see that for households not particularly dependent on pension income, there was no drop in consumption, regardless of the period covered by the estimation sample. In contrast, there were substantial expenditure drops in households that depended on pension income, and especially during the MoU period. Although not reported here, we found no expenditure drops for retirees collecting pensions above the median of the monthly pension distribution, i.e., pensions higher than €1323 per month. The effect was observed during turbulent times for those receiving a monthly pension lower than €1323.

**Table 6:** Retirement and expenditure: Results by dependency on pension income. *Source:* Household Budget Survey (2008–2018); Hellenic Statistical Authority (EL.STAT)

	Total period	Before MoU (2008M1–2010M5)	During MoU (2010M6–2018M12)
	[1]	[2]	[3]
Panel A: Unconditional on household income			
Retirees with low dependency on pension income	− .585 (.701)	.171 (1.263)	− .319 (.261)
Observations	7207	1566	5641
Retirees with high dependency on pension income	− .413* (.223)	− .176 (.774)	− .408* (.198)
Observations	7935	1526	6409
Panel B: Conditional on household income			
Retirees with low dependency on pension income	− .127 (.337)	− .269 (.668)	− .047 (.166)
Total household income	.630*** (.049)	.657** (.094)	.624** (.026)
Observations	7207	1566	5641
Retirees with high dependency on pension income	− .358* (.176)	.229 (.608)	− .340* (.156)
Total household income	.625*** (.015)	.634*** (.031)	.626*** (.017)
Observations	7935	1526	6409

2SLS estimates. Retirees with low dependency on pension income are those with a dependency ratio (total pensions to total household income) lower than 0.68. Robust standard errors in parentheses. All models include individual and household controls and region and year fixed effects

^†^p < .1. *p < .05. **p < .01. ***p < . 001

What is more interesting is that the observed drop in consumption (during the austerity period for retirees with a high dependency on pension income) remained after controlling for total household income, as seen in Panel B. Controlling for variation in income did explain part of the drop; however, the estimated coefficient remained negative and sizeable.

Consistent with the predictions of the life-cycle model which assumes away large income shocks, our results suggest that retirees did not reduce their expenditure in good times. This also holds for the early years after the first MoU was signed, during which a number of pension cuts were applied. In an earlier discussion, we attributed this finding to the fact that the first cuts were relevant for retirees below 60 years old (and receiving relatively high pensions). If an income shock mechanism for certain groups was in operation, then those groups should have been affected by the cuts during that early crisis period. As discussed earlier, these effects could not be reflected in our baseline estimates given the relatively small size of those groups, i.e. young retirees. However, to empirically test for this hypothesis, we performed the following exercise. We collapsed by age, year, quarter, and region the mean pension income, total income, and expenditure of retirees. This was done for the period 2010Q1–2013Q4; i.e., the early crisis period during which young retirees saw their pensions cut. We considered quarters 2011Q4 onward to be the post-policy period. Retirees up to 60 years old were the treated group and those above 60 were the control group.

In Table 7, we report estimates specific to the MoU period. There was a significant drop in work-related expenses for those with high dependency on pensions, while this impact on expenditure for necessity goods and expenditure for non-necessity goods was lower in terms of magnitude and significance. The effect on work-related expenditure for retirees with low dependency on pension income was negative but weak. These results suggest that retirees characterized by high dependence on pensions reduced their work-related expenditures, as well as their expenditures for necessity and non-necessity goods during the reforms period. Therefore, not all sorts of expenditure declines should be seen as necessarily leading to loss of quality of life, especially when considered under more general frameworks, e.g., retirees turn to healthier and home-prepared meals, they consume less energy due to cutting down commuting to work, and they are less exposed to public health threats due to various pathogens. On the other hand, retirees with low dependency on pensions did not suffer a sizeable drop in any expense category. Hence, it is important to distinguish between retirees when looking into expenditure declines in order to design targeted interventions in order to help those retirees who face specific adverse shocks, e.g., retirees highly dependent on pensions who cut down on necessity goods consumption.

**Table 7:** Retirement and expenditure categories during the reforms period (2010M6–2018M12). *Source:* Household Budget Survey (2008–2018); Hellenic Statistical Authority (EL.STAT)

	Food & beverages	Work-related expenses	Expenses for necessity goods	Expenses for non-necessity goods
	[1]	[2]	[3]	[4]
Retirees with high dependency on pension income	− .120 (.165)	− 1.580** (.596)	− .322† (.167)	− .837* (.330)
Observations	6409	6409	6409	6409
Retirees with low dependency on pension income	.030 (.172)	− 1.119† (.680)	− .073 (.192)	− .782 (.481)
Observations	5641	5641	5641	5641

2SLS estimates. Robust standard errors in parentheses. All models include individual and household controls and year fixed effects

^†^p < .1. *p < .05. **p < .01. ***p < . 001

Table 8 displays the difference-in-differences (DiD) results for three outcomes: pension, total income, and expenditure. The treated group coefficient is positive and significant, which confirms that those retirees were collecting high pensions. The DiD estimates are everywhere negative and significant, which indicates that young retirees were affected relatively more once the first cuts started being implemented.

**Table 8:** Difference-in-differences results. *Source:* Household Budget Survey (2008–2018); Hellenic Statistical Authority (EL.STAT)

Dependent variable	Treated group (ages ≤ 60)	Post period (after 2011Q3)	Treated X Post (DiD estimate)	Observations (sample: 2010–2013)
	[1]	[2]	[3]	[4]
Pensions	0.220*** (.001)	− 0.020*** (.001)	− 0.056*** (.001)	829
Total income	0.248*** (.001)	− 0.048*** (.001)	− 0.154*** (.001)	841
Expenditure	0.266*** (.001)	− 0.045*** (.001)	− 0.064*** (.001)	841

Dependent variable	Treated group (dependency ratio ≥ 0.68)	Post period (after 2014)	Treated X Post (DiD estimate)	Observations (sample: 2011–2017)
Pensions	0.236*** (.001)	− 0.136*** (.001)	− 0.144*** (.001)	1520
Total income	− 0.072*** (.001)	− 0.160*** (.001)	− 0.106*** (.001)	1527
Expenditure	− 0.150*** (.001)	− 0.130*** (.001)	− 0.004*** (.001)	1527

Poisson regression estimates. Outcomes are in levels. Observations are age-region-year-quarter cells for retirees 10 years around the ERA. Retirees with low dependency on pension income are those with a dependency ratio (total pensions to total household income) lower than 0.68. Robust standard errors in parentheses. All models include individual and household controls and region and year fixed effects

^†^p < .1. *p < .05. **p < .01. ***p < . 001

Just as retirees below 60 years old were the treated group during the first turbulent years, those who were heavily dependent on pensions can be considered to be the treated group throughout the period during which the pension cuts were being implemented—and especially after 2014, when pension cuts became horizontal. In this case, and using the collapsed dataset from above, we considered ages for which the pension dependency ratio was equal to or above 0.68 as the treated group; those for which the ratio was lower formed the control group. The period after 2014 was the policy-on period and the sample covered the period 2011–2017. The lower panel of Table 8 reports the DiD parameter estimates. Again, the interaction terms were negative and significant, which supports the decline in expenditure through an income mechanism during that period.

**Fig. 9:**
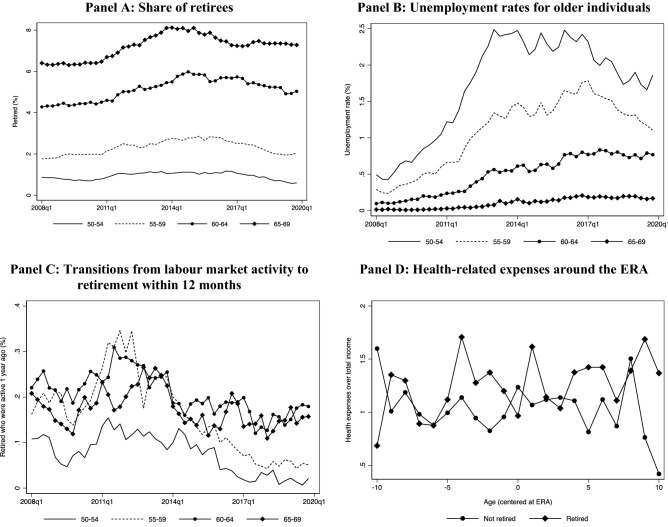
Labour market and health expenditure trends for individuals close to retirement. *Source:* Labour Force Survey (2008–2018); Hellenic Statistical Authority (EL.STAT). *Notes:* Shares are weighted by the sampling weights. In all graphs the denominator is the total number of labour-market-active individuals (employed or unemployed) aged 15–74 years

Therefore, our evidence favours a drop in consumption at retirement during the austerity period, and this is more pertinent for those heavily dependent on pension income. This can be attributed to the implementation of several pension cuts and reforms that took place during that period. However, while economic conditions deteriorate, such negative income changes may not be merely due to institutional changes but also to adverse individual labour market outcomes. According to EL.STAT press releases, the overall unemployment rate was oscillating around 27% between mid-2012 and the first quarter of 2015. Hence, a substantially increased pool of unemployed around retirement age could weaken our argument about a reforms-based mechanism. We were not able to formally test how individual labour market shocks affected the observed drop, because the HBS data do not follow individuals and their households over time. However, we used individual-level data from the quarterly LFS for the period from 2008 onward to document some labour market trends around the ERA. Figure 9 presents the results.

Relative to the total labour-market-active population aged 15–74 years, the shares of retirees in age groups around the ERA were rather stable when considering those 50–59 years old (panel A). There was a small increasing trend from 2011 to 2015, but mainly for those older than 65 years. At the same time, the unemployment rate for older age groups increased, especially for those 50–54 years old, rather than those closer to the ERA (panel B). Also, the shares of those who retired but were active in the labour market 12 months earlier appeared to increase, during 2011–2013, for all age groups around the ERA. However, the numbers were rather small to account for the overall effect (panel C).^11^ The shares were even smaller and noisier if we retain in the denominator only retirees who were unemployed 12 months earlier. Therefore, the worsening labour market conditions observed for those around the ERA were not very likely to explain the drop. Finally, using HBS data on expenditures, health-related expenses—as a share of the household’s total monetary income—were low and rather stable around the ERA. Hence, we would rather exclude significant health-related shocks as a driver of our results (panel D). If anything, our baseline estimates should be interpreted as lower bound estimates of the true effect of retirement on consumption for those who are mostly dependent on pensions during uncertain times.

## Conclusions

Despite the fact that expenditure changes at retirement have been extensively investigated, little is known about how this relationship behaves during economically turbulent periods. In this paper we fill this gap. Using Greek data for the period 2008–2018, we observe some interesting patterns regarding household expenditure around retirement during the financial crisis period that was signified not only by an economic downturn but also by the implementation of significant pension reforms. This offers an ideal setting to evaluate the effect of retirement on household expenditure during turbulent times and compare it with normal times, because unexpected negative pension shocks might lead to revisions of expected future pension wealth. To address the endogeneity of individual retirement status, we exploit the retirement legislation in Greece in an instrumental variables framework in which the retirement probability is higher for individuals close to the early retirement age threshold. Within this estimation framework, we use the early retirement eligibility threshold to predict individual retirement status.

Our 2SLS estimates when using data from the total period suggest that head of household retirement is associated with a drop of 18.5% in total household consumption on average. Going further, we perform several cuts to the data to see where the effect comes from. According to our results, our baseline estimates are driven by the later (MoU) sub-period, which was intensive in terms of pension reforms and cuts, and particularly by pensioners with a high dependency on income from pensions. No evidence for a significant drop in household expenditure was found during normal times for either retiree type. Also, there were no significant drops in consumption for pensioners who do not depend heavily on income from pensions, regardless of the period our models are estimated for.

Our results also suggest a gender asymmetry. Total household expenditure drops significantly when the husband retires and as he grows older, but there are no level or age-related effects regarding wife’s retirement. One possible explanation, which is discussed in the literature, is that that the wife is often the second earner in the household and therefore her retirement affects the household less than her husband’s (Moreau & Stancanelli, 2015) or because she is typically younger than the husband by the time she reaches retirement age, therefore, the household has adjusted to income changes. It should be noted that our empirical strategy and findings have certain limitations. First, the estimation sample before the MoU period is considerably smaller relative to the sample during the MoU period. This leads to a noisy estimate of the retirement impact for the former period which does not seem to be statistically significant from the estimate regarding the period during the MoU implementation. Second, although our data come from a 10-year rotating panel, individual and household longitudinal identifiers were not available, thus restricting us from pursuing an analysis that would exploit within-unit time variation, such as an instrumented difference-in-differences approach. Finally, our analysis focuses on expenditure changes at retirement but does not account for changes in quality of life. Whether certain choices of individuals after retirement improve health outcomes or quality of life could not be explored in our study and requires further data. However, it is an important thing to consider because consumption declines at retirement should not be considered as necessarily leading to quality of life losses. For example, retirees are less time constrained and therefore they can substitute harmful health habits (such as eating out or following a less considerate diet given their working schedules) for the production of healthier, home-cooked meals. The unavoidable reduction in their work-related expenses as that was the case with those highly dependent on their pension in our sample, e.g. commuting to work, is not necessarily bad either, especially when examined under a more general framework of fighting climate change and increase energy saving, or even from a public health perspective in the sense that less commuting means less exposure to potential health threats such as the SARS-CoV-2 virus, which led to the COVID-19 pandemic, or other pathogens.

Overall, our study documents that only vulnerable pensioners suffered a significant drop in their household expenditure during a turbulent period, and provides no evidence of a puzzle during normal times. This is important because financial hardship, expected changes in future income and various liquidity constraints are major determinants of individual and household behaviours (Fan et al., 2022; Moreno-Herrero et al., 2017). As seen in previous studies, those above 65 years old were also those who were affected the most in terms of mortality and health outcomes in the onset of the financial crisis in Greece (Laliotis & Stavropoulou, 2018; Laliotis et al., 2016). Considering that household consumption forms a substantial proportion of the overall GDP, and given the country’s aging profile, our findings have important policy implications concerning pension reforms that are planned during financial crises. These are expected to impact not only retirees themselves, and especially those the most dependent on income from pensions, but also their household expenditure in total. Our findings suggest that pension reforms during periods of financial crisis must protect not only those with low pensions but also, and crucially, those whose households depend more on pensions and do not have other sources of income. This is important to ensure that retirement is not associated with quality of life losses, and any interventions should be targeted to support individuals getting hit by specific adverse shocks, e.g. retirees highly dependent on pensions who cut down on necessity goods consumption. Given the country’s ageing profile, our results have important implication for policies targeting not just a growing part of the population, but also a part for which there is evidence that has the potential to remain productive and engaged in prosocial activities even after retirement (Dosman et al., 2006; Georganas et al., 2022).

## Data Availability

The data for this study were made available after an agreement with the Hellenic Statistical Authority (ELSTAT). The public use version of the data is available on www.statistics.gr.

## Footnotes

1 Auxiliary pensions are financed separately from main pensions by both employers and employees, without any state contribution, and all pensioners receiving a main pension are entitled. For more details on the structure of the Greek pension system, please go to this link.

2 More specifically, main pension cuts ranged from 3% (for main pensions higher than €1400) to 13% (for main pensions higher than €3500). Auxiliary pension cuts ranged from 3% (for auxiliary pensions higher than €300) to 10% (for auxiliary pensions higher than €650).

3 A similar rationale holds for omitting cohabiting couples from the sample. Excluding cohabiting and same-sex couples from our sample results in 22 fewer observations in total, i.e. our utilized sample allowing for 15 years on each side of the early retirement age threshold would be 10,075 observations instead of 10,053. This small number of observations can be due to (a) the framework regulating cohabitation and same-sex marriage at the time, and (b) the fact that although cohabitation and same-sex marriage is becoming more common options for younger couples, our empirical strategy considers a specific age bracket around retirement, hence, individuals are of considerably older age. Moreover, there is no solid information to ensure that those 22 observations refer to households where the head and the partner are actually a couple and behave accordingly.

4 Earlier LFS data are not suitable because age was recorded in 5-year bands.

5 Moreover, individuals who tend to retire considerably before ERA come from very specific sectors of the economy. Using LFS data, they are more likely to come from sectors such as Mining and Quarrying, as well as Electricity and Water Supply, which are dominated by the public sector.

6 Our baseline results remain unchanged when including unmarried cohabiting and same-sex couples in our sample, resulting in increasing the sample by 22 observations, i.e. 10,075 in total. More specifically, the baseline coefficient of household head retirement is − 0.202 (standard error = 0.089), and the baseline coefficient of partner retirement on household consumption is − 0.024 (standard error = 0.129). First-stage results also remain unchanged. Crossing the ERA threshold increases the probability of retirement by 21.2% (standard error = 0.018) for household heads. For their partners, becoming eligible for early retirement increases the probability of retirement by 13.6% (standard error = 0.019).

7 Halvorsen and Palmquist (1980) suggest that in semi-logarithmic models the interpretation of coefficients of binary variables coefficients should be made using the formula $100\times [{e}^{\beta }-1]$, where *β* are the estimated effects from Eqs. (1) and (4).

8 Following a stacked regression approach, we failed to confirm that the retirement coefficients for the samples before and during the MoU period are statistically significant from each other at the 10% level. This could be due to the lack of statistical power as the sample from before the MoU period is considerably lower than the during the MoU period one. However, there is still evidence indicating that behaviour around retirement changed considerably between the two periods, i.e. there is a sizeable difference in the first-stage results between the two sub-periods (Table 2), at least for household heads.

9 Moreover, 91.3% of respondents stated that when they started to receive their main or supplementary pensions, it was not due to already established early pension rights.

10 Results available upon request.

11 We consider retirees to be those who are not active in the labour market (either as employed or unemployed), identify themselves as pensioners, and report that an old-age pension (rather than a disability pension) was their main income source in the current quarter.
